# Response to: Comment on ‘Comparing specificity and seroconversion sensitivity among major blood screening assays for human immunodeficiency virus and viral hepatitis’

**DOI:** 10.1111/vox.70323

**Published:** 2026-09-08

**Authors:** Monica Chaves, Martina Schaetzl, Florina Pessl

**Affiliations:** ^1^ Roche Diagnostics International Rotkreuz Switzerland; ^2^ Roche Diagnostics GmbH Penzberg Germany

We thank you for the opportunity to respond to the Letter to the Editor by Sepetiene et al. [1] regarding our original article ‘Comparing specificity and seroconversion sensitivity among major blood screening assays for human immunodeficiency virus and viral hepatitis’.

The aim of our publication was to highlight the importance of an assay with the best possible specificity and seroconversion sensitivity in blood donor screening to ensure the highest possible blood safety. Sepetiene et al. questioned the validity of comparing antibody‐only (Ab‐only) assays with an antigen/antibody (Ag/Ab) combination assay for hepatitis C virus (HCV). However, Ag/Ab combination assays offer unprecedented early detection capabilities compared with Ab‐only platforms, and a direct comparison between these methodologies enables a robust assessment of this diagnostic improvement, as documented in previous studies [2].

This paradigm shift is already well established in human immunodeficiency virus blood donor screening, where Ag/Ab combination immunoassays have largely superseded Ab‐only screening tests due to their demonstrated ability to improve the detection rate and reduce the turnaround time for results [3, 4, 5, 6], a trend further supported by multicentre performance evaluations [7].

In this context, we believe that the argument that comparator assays must have identical intended uses in terms of assay architecture is not relevant. All evaluated immunoassays, whether Ag/Ab combination or Ab‐only, share the fundamental intended use of screening blood donations to prevent transfusion‐transmissible infections. The scientific and transfusion medicine communities prioritize the implementation of assays that provide the best possible diagnostic performance for blood screening, regardless of the underlying biomarker detection concept.

We also contest the claim that our study design lacked the rigour of a head‐to‐head comparison. The references cited by Sepetiene et al. [6, 7, 8] primarily address quantitative assays and evaluations with limited sample sizes, which do not apply to our study. To minimize pre‐analytical variability, we carefully aliquoted seroconversion panels prior to testing and distributed individual aliquots to the respective testing sites, adhering to standard laboratory procedures. The samples were not transported between sites after measurement. Furthermore, we tested the specificity cohort at a single site using the same operators and consistent environmental conditions, which minimized variability. As noted in the limitations section of our article, the varying time intervals between sample draws within the seroconversion panels are an unavoidable constraint of commercial panels. This variation only impacts the calculation of the average time interval from nucleic acid testing (NAT) positivity to assay detection.

Regarding data presentation, the use of percentage shifts (e.g., earlier, equal or later detection) to report seroconversion sensitivity was a deliberate choice to provide a clear, comprehensive overview of assay performance across the 183 evaluated seroconversion panels. Detailing the specific day‐by‐day differences for every single panel would have made the results impractical for readers to follow and led the article to significantly exceed the journal's word limit.

A description of the monoclonal antibody design of the Elecsys HCV Duo assay was outside the scope of this study, as this information has been comprehensively described in previous literature [8]. Finally, we agree that the exclusive use of seroconversion panels originating from the United States restricts the applicability of our analysis to a narrow range of genotypes, and we have explicitly acknowledged this inevitable limitation in our publication. However, the current lack of commercially available seroconversion panels from diverse geographical regions does not invalidate the broad genotype and serotype recognition of the Elecsys assays, which has been extensively demonstrated in other clinical evaluations [8, 9, 10, 11]. We plan on conducting further studies with seroconversion panels from diverse geographical regions once they become available.

## CONFLICT OF INTEREST STATEMENT

F.P., M.C. and M.S. are employees of Roche, the sponsor of this study that was already published in *Vox Sanguinis*. F.P. and M.C. hold stocks/stock options with Roche.

## Data Availability

Reasonable requests for datasets generated during and/or analysed during the current study may be directed to rotkreuz.datasharingrequests@roche.com.
